# The Leadership of the Brazilian Journal of Cardiovascular Surgery in the Digital Transformation of Cardiovascular Surgery

**DOI:** 10.21470/1678-9741-2025-0454

**Published:** 2026-02-18

**Authors:** Vivian Masutti Jonke, Henrique Murad, Andre Luppi

**Affiliations:** 1 Universidade Federal de São Paulo, São Paulo, São Paulo, Brazil; 2 Universidade Federal do Rio de Janeiro, Hospital Universitário Clementino Fraga Filho, Rio de Janeiro, Rio de Janeiro, Brazil; 3 Massachusets General Hospital, Harvard Medical School, Boston, Massachusetts, United States of America

In recent decades, the incorporation of advanced digital technologies has profoundly transformed the way medicine is practiced, researched, and taught. Artificial intelligence (AI), telemedicine, big data, the Internet of Things (or IoT), robotics, and 3D printing, terms that authors refer to as “Medicine 5.0”, have moved beyond the boundaries of research centers to become increasingly integrated into the clinical routine of multiple medical specialties to advance precision medicine^[1,2]^.

However, only a decade ago, the idea of these technologies becoming part of daily cardiovascular surgery practice was unlikely. Technological infrastructure was concentrated in a few centers worldwide, and technologies such as AI were largely restricted to specialties with a high volume of structured and standardized diagnostic data such as radiology, pathology, dermatology, ophthalmology, and cardiology, without properly addressing the complexity of the surgical environment^[2]^.

This scenario has changed rapidly. The dissemination of these technologies into surgical fields, particularly AI, was initially modest, limited to experimental initiatives and isolated research efforts. Two factors, however, catalyzed a broader transformation. The first was the coronavirus disease 2019 (or COVID-19) pandemic, which forced the medical and scientific community to process large volumes of data in real time, with both speed and quality, to support critical clinical and public health decisions^[3]^. The second was the diffusion of generative AI into personal devices, democratizing access to advanced computational tools and creating favorable conditions for large scale adoption (Figure 1).

**Fig. 1:**
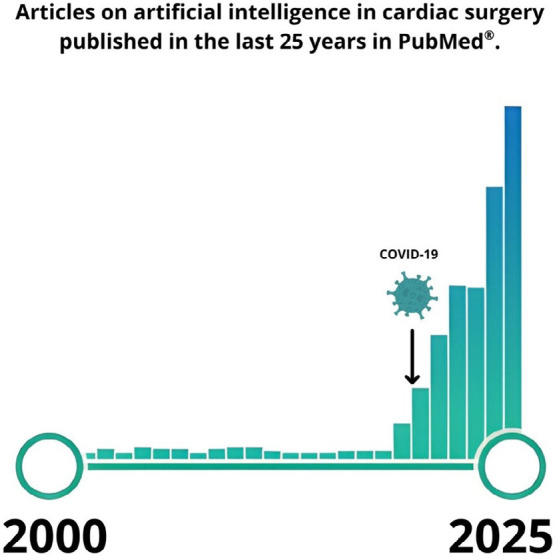
Trend of PubMed® publications combining the terms “artificial intelligence” and “cardiac surgery” or “cardiothoracic surgery” from 2000 to 2025. Late 2019 and early 2020 are highlighted, marking the beginning of the coronavirus disease 2019 (COVID-19) pandemic. Search strategy: (“artificial intelligence” AND [“cardiac surgery” OR “cardiothoracic surgery”]). Source: https://pubmed.ncbi.nlm.nih.gov/

This convergence of clinical need and technological accessibility paved the way for a more structured integration of AI and other digital technologies into surgery. Cardiovascular surgery, in particular, is well positioned to benefit from these advances. The integration of machine learning algorithms, predictive models, and three-dimensional visualization technologies can support more precise and timely decision-making, enhance surgical planning, improve risk stratification, and optimize perioperative management. Furthermore, it is essential to discuss widely how these technologies are being incorporated into clinical practice and scientific production, including their legal, ethical, and regulatory aspects, which may have direct affect, positively or negatively, on the quality of biomedical research.

Medicine 5.0, characterized by the integration of AI, automation, robotics, and advanced data analytics, is consolidating itself as a new paradigm. This transition does not replace the expertise of the cardiovascular surgeon but rather expands it, providing analytical tools capable of identifying complex patterns, predicting events, and optimizing care pathways. As in other specialties, the introduction of AI in surgery raises questions about the role of the human professional. While the manual and decisional nature of surgery makes replacement unlikely, one principle remains: those who master these technologies will probably have a strategic advantage. It reinforces the central role of the cardiovascular surgeon as the conductor of critical decisions, now supported by tools that expand their analytical capacity. Clinical expertise remains irreplaceable but now operates in a more sophisticated ecosystem, where data and algorithms are strategic allies.

For this integration between surgeons and new technologies to occur effectively, ethically, and sustainably, more than access to tools is required. Digital proficiency is essential, meaning the technical and conceptual mastery needed to understand, use, and critically assess these solutions. This proficiency ranges from basic operational skills to advanced knowledge of data governance, information security, algorithmic bias, and ethical, as well as legal, implications.

The application of AI in scientific research is already tangible. In cardiovascular surgery, predictive models and analytical tools are being used for surgical risk stratification, the creation of three-dimensional models, and the development of digital twins, enabling preoperative simulations with a potential impact on patient safety and procedural precision^[4,5]^. Additionally, generative AI tools are increasingly used to support scientific writing, article curation, and literature interpretation. Today, the central question is no longer whether to use AI but how to use it, what are the limits and safeguards, to ensure the quality, integrity, and reliability of scientific output^[6]^.

The adoption of these technologies is not uniform: institutions, regions, and professionals are at different levels of digital maturity. The historical absence of these topics from medical curricula highlights a gap that must be addressed. Digital transformation represents a cultural shift in how medical knowledge is produced, transmitted, and applied. For this transition to succeed, collaboration between generations is essential. The clinical expertise of senior professionals, combined with the technological fluency of younger surgeons, offers a unique opportunity for synergy.

In this context, the Brazilian Journal of Cardiovascular Surgery (BJCVS) plays a strategic role with the responsible lead of that process, promoting education, debate, and the dissemination of these technologies among cardiovascular surgeons, researchers, and trainees in Brazil^[7]^. Recent examples include the 2024 and 2025 Sociedade Brasileira de Cirurgia Cardiovascular (SBCCV) Meetings, which featured focused discussions on AI and innovation, as well as editorials, the appointment of associate editors with expertise in the field, the encouragement of scientific submissions, and courses such as Cardiovascular Surgery 5.0. These initiatives place BJCVS at the forefront of the digital transformation debate in the specialty, providing training frameworks, ethical standards, and forums for qualified debate turning abstract concepts into practical, applicable experiences.

BJCVS plays a distinctive role not only by disseminating technical knowledge but also by acting as an agent of transformation. By opening space for discussions on these new technologies, it contributes to positioning Brazilian cardiovascular surgery at the center of global debates on the future of medical practice^[3]^.

For this transformation to be sustainable, it is essential to invest in continuous training, the development of protocols for responsible use, and the rigorous evaluation of the scientific evidence supporting each tool. Medical ethics and patient safety remain core principles, regardless of technological advancement. The leadership of Brazilian Journal of Cardiovascular Surgery in this process is reflected not only in content dissemination but also in the establishment of guidelines, the promotion of qualified debates, and the encouragement of applied research, just as it has historically occurred with transformative innovations such as countless surgical techniques, improvements in cardiopulmonary bypass circuits, prosthetic surgical and transcatheter valves, and endovascular aortic stent grafts, among others.

AI and digital technologies represent a frontier that is no longer future but present. The choice facing the cardiovascular surgical community is clear: adapt to and lead this transformation or be led by it. By explicitly embracing its editorial and scientific responsibility and quality in this field, BJCVS reaffirms its strategic role in building a more precise, integrated, and forward-looking cardiovascular surgery, positioning itself at the forefront of global surgical journals.

## Artificial Intelligence Usage

The authors declare use of ChatGPT for language editing. The content produced by the artificial intelligence tool was revised and edited by the authors as necessary, and they take full responsibility for the content to be published.
